# Coronary Artery Dilation in Children with Febrile Exanthematous Illness without Criteria for Kawasaki Disease

**DOI:** 10.5935/abc.20190191

**Published:** 2019-12

**Authors:** Jesus Reyna, Luz Marina Reyes, Lorenzo Reyes, Freya Helena Campos, Patricia Meza, Alfredo Lagunas, Carla Contreras, Ana Elena Limón

**Affiliations:** 1 Hospital Central Sur de Alta Especialidad - Pediatria, Ciudad de México - México; 2 Hospital Central Sur Pemex - Pediatria, Ciudad de México - México; 3 Hospital Central Sur Pemex - Cardiología, Ciudad de México - México; 4 Hospital Central Sur Pemex - Alergología, Ciudad de México - México; 5 HCSAE Pemex, Ciudad de México - México; 6 INSP México, Ciudad de México - México

**Keywords:** Child, Coronary Disease, Evanthema, Fever, Kawasali Disease, Mucocutaneous Lymph Node Syndrome, Echocardiography/methods

## Abstract

**Background:**

Coronary dilatation is the most important complication of Kawasaki disease (KD) and, in addition to some clinical characteristics, is common to KD and febrile exanthematous illnesses (FEIs).

**Objective:**

To assess whether children with FEI, who do not meet the criteria for KD, have changes in coronary arteries dimensions.

**Methods:**

Echocardiography was performed within the first two weeks of the disease in patients < 10 years with fever and exanthema without other KD criteria. To make a comparison with KD patients, we reviewed the echocardiograms and medical records of patients with a diagnosis of KD of the last five years. Coronary ectasia was assessed using Z scores of coronary arteries. The means of the dimensions of the coronary arteries were compared with a z test and a level of significance of 0.05 was adopted.

**Results:**

A total of 34 patients were included, 22 (64.7%) with FEI, and 12(35.2%) with a diagnosis of KD. Using the Z scores of coronary artery, a dilation of any of the coronary artery branches was observed in six (27.2%) patients with FEI.

**Conclusions:**

An important percentage of patients with FEI has coronary artery dilation.

## Introduction

Up to some years ago, exanthema and fever in children were diagnosed as one of the diseases of the complex known as febrile exanthematous illnesses (FEI), including measles, rubella and scarlet fever. Thus, it was considered that, in most cases, symptoms would disappear by symptomatic treatment.^1^ As vaccination schedule became universal, the epidemiology of FEIs has changed in a way that Kawasaki disease (KD), which was once an exception among these diseases, has become the primary illness to be considered in face of clinical signs including persistent fever and exanthema. Coronary abnormalities are the most serious complications of KD.^2,3^

Besides fever and exanthema, FEIs and KD share other clinical characteristics, such as conjunctival injection, swollen lymph nodes, and, in some cases, desquamation and swelling of the limbs, which supports the suspicion of KD in any of its forms.^4,5^ It is paradoxical that, when the clinical presentation of incomplete KD is confused with self-limited diseases such as FEIs, the occurrence of a serious cardiovascular disease as a complication may be neglected.^6^

Studies have established that FEIs and KD have in common pathophysiologic mechanisms and clinical signs,^7^ and therefore, some infectious agents have been proposed as responsible for causing KD. This implies that patients that have been diagnosed with FEIs without meeting the criteria for KD could develop coronary abnormalities.^7,8^ These are considered an uncommon cause of cardiac disease among pediatric patients. However, the ensuing mortality, in some cases, makes them relevant in clinical practice.^9^

Given all of this, we intend to assess whether children with febrile illness who do not meet the criteria for KD have changes in coronary arteries dimensions.

## Methods

In a cross-sectional study, we included patients under 10 years of age with a diagnosis of FEI in the pediatric outpatient settings of two hospitals belonging to the Health Services of Petróleos Mexicanos in Mexico. The parents signed an informed consent to participate in the study. The American Heart Association (AHA) criteria^10^ were applied to all cases to confirm that the KD diagnostic criteria were not met, including cases that could be considered atypical or incomplete. All the patients underwent an echocardiogram within the first two weeks of the disease using a Vivid 7 General Electric® device.

Patients who had a fever ≥ 38°C, which had lasted for at least one day, and exanthema were considered. Subjects with previous known diseases, such as arterial hypertension, family history of cardiopathy, congenital cardiopathy, children with weight above the 95th percentile or below the 5th percentile according to their age, or who had been using steroids during at least one month before the disease were not included.

To establish a comparison with KD patients, we reviewed the echocardiograms and the medical electronic records of patients with a diagnosis of KD detected by the Pediatric Cardiology Service during the last five years.^11^ Those who met the AHA criteria for KD were included in the analysis, and the children were then selected by convenience sampling.

### Assessment of the coronary arteries

Echocardiography was performed according to that described by Muniz et al.^12^ Coronary ectasia (CE) was defined as a dilation of the coronary artery > 1.5-fold in diameter, evidenced by an echocardiogram, when compared to the adjacent normal segments of the same arteries according to coronary artery Z scores.^11^ The mean Z score for each coronary artery segment was 0, with a SD of 1.

### Statistical analysis

Comparison of the means of the dimensions of the coronary arteries was performed with a z test, using a one-tailed analysis with a 95% confidence interval. Demographic and clinical characteristics were analyzed using an unpaired Student’s t-test or a Fisher’s exact test, depending on the type of variable, with a difference of p < 0.05. No adjustments were performed in the analysis, since the intention of the study is exploratory. The analysis of the results was done through the Stata program version 13.

### Ethics

The study was approved by the Research and Ethics Committees at both hospitals. Informed consent signed was signed by the parents of the children included in the study.

## Results

We included a total of 34 patients: 22 (64.7%) had a diagnosis of non-KD FEI; 11 of them (50%) were diagnosed as viral exanthema, the most common were hand-foot-and-mouth disease (n = 5; 22.7%) and exanthema subitem (n = 4; 18.1%); there was also one case of scarlet fever and one of Gianotti-Crosti syndrome. The remaining 12 patients were diagnosed with KD.

Distribution by sex was as follows: in the FEI group, 13 (59%) patients were male and nine (40.9%) were female; in the KD group, nine (75%) patients were male and three (25%) were female. In terms of age, in the FEI group, the mean age was 41.3 months (range: 7 to 120 months), and in the KD group, the mean age was 18.1 months (range: 6 to 36 months). Other demographic and clinical variables are described in Table 1.

**Table 1:** Demographic and clinical characteristics of patients with febrile exanthematous illness (FEI) and patients with Kawasaki disease (KD)

Variable	FEI (n = 22)	KD (n = 12)	p value
Male (%)	59	75	0.02
Age, in months (mean)	41.3	18.1	0.05
Fever duration, in days (mean)	3.6	6.5	0.06
Maximum body temperature (°C)	38.3	38.7	0.9
Exanthema	22(100)	12 (100)	1
Conjuntivitis	2(6.2)	12 (100)	0.03
Swollen lymph glands in the neck	0(0)	12 (100)	--------
Swelling and redness in hands and bottoms of feet, n (%)	0(0)	12 (100)	--------
Peeling skin n (%)	0(0)	12 (100)	-----------
Swollen tongue n (%)	0(0)	12 (100)	-----------
Time between diagnosis and echocardiography, in days (mean)	12	25.3	0.05
Coronary dilatation n (%)	6 (27.2)	4(33.3%)	0.4

### Kawasaki disease criteria

Regarding the diagnostic criteria for KD in our sample, we found that the average duration of fever was 3.6 ± 2 days, only six subjects (27.2%) met the fever duration criterion of ≥ 5 days. The average of body temperature peak was 38.3°C.

Exanthema was present in all subjects, since it was one of the inclusion criteria in the study. One of them had conjunctival hyperemia; none of them had edema, desquamation of foot, hand or tongue, or swollen ganglia. The comparison with the KD patients is shown in Table 1, which shows a higher frequency of some clinical problems in children with KD, swollen lymph glands in the neck, swelling and redness in hands and bottoms of feet, peeling skin and swollen tongue. No difference was found in these percentages compared with those of patients with coronary dilation.

### Assessment of coronary arteries in subjects with FEI

Measurements of the left main coronary artery (LMCA), proximal right coronary artery (PRCA), medial right coronary artery (MRCA), distal right coronary artery (DRCA), circumflex, left anterior descending coronary artery (LAD) were available in 22 patients with FEI. The PRCA showed the largest dilation (mean Z score = 0.45 ± 0.63, p < 0.005), followed by the LMCA (mean Z score = 0.14 ± 1.0, p < 0.05) (Table 2). According to the coronary artery Z-scores, six (27.2 %) patients diagnosed with FEI showed dilation in at least one of the coronary branches. Comparison between the groups are shown in Table 3.

**Table 2:** Z scores of coronary arteries of subjects with febrile exanthematous illness and dilatation of at least one of the coronary branches

Gender	Age (Months)	PRCA	Z	MRCA	Z	DRCA	Z	LMCA	Z	Circumflex	Z	LAD	Z	Diagnosis
F	7	2	*1.7	1.9	*2.1	1.6	1.57	3	*4.1	2	*2.5	2.8	*4.9	SE
F	27	2.4	*1.67	1.7	0.51	1.4	-0.1	2.2	0.49	1.6	0.29	1.5	-0.16	HFM disease
M	84	3	*2.21	1.9	0.27	1.5	-0.53	3.3	*2.3	1.7	-0.16	1.6	-0.6	Scarlet fever
M	120	3.4	*1.85	3	*1.7	2.3	0.47	3.5	1.48	2.6	0.94	2.5	0.74	Viral exanthem
F	36	2.3	1.16	2	1.09	1.8	0.82	3.1	*2.6	2.1	1.4	2	1.07	HFM disease
M	36	1.6	-0.57	1.2	-0.92	1	-1.34	2	-0.2	1.1	-1.16	1	*1.7	HFM disease
Mean	51.6	2.45	0.93	1.95	0.24	1.6	0.15	2.85	0.59	1.85	0.26	1.9	0.26	xx

* Increased Z scores; F: Female; M: Male; PRCA: Proximal Right Coronary Artery; MRCA: Medial Right Coronary Artery; DRCA: Distal Right coronary artery; LMCA: Left main coronary artery; LAD: left anterior descending coronary artery; Z: Z values; SE: Sudden exanthema; HFM: Hand Food Mouth

**Table 3:** Comparison of Z scores of coronary arteries between subjects with febrile exanthematous illness and subjects with Kawasaki disease

	FEI (mean(IC 95%)	KD	p
PRCA	0.45 (-0.01-0.9)	0.2	0.05
MDCA	-0.004 (-0.3-0.3)	4.8	0.05
DRCA	-0.2(-0.8-0.3)	2.3	0.05
LMCA	0.13 (-0.2-0.5)	0.6	0.05
Circumflex	-0.01(-0.4-0.4)	0.6	0.05
LAD	-0.36 (-0.01-0.5)	0.5	0.05

PRCA: Proximal Right Coronary Artery; MRCA: Medial Right Coronary Artery; DRCA: Distal Right coronary artery; LMCA: Left main coronary artery; LAD: left anterior descending coronary artery.

## Discussion

Previous publications have reported cases of increase in the dimensions of the coronary arteries among subjects with diseases such as polyarteritis nodosa, periodontal disease, Mediterranean spotted fever caused by *Rickettsia*, murine typhus, and even rheumatic fever. Furthermore, it has been shown that the dimensions of the coronary arteries of children with prolonged fever, who do not meet the criteria for KD, are larger than those of healthy subjects, but smaller than those of children suffering from KD.^12-15^ These findings are in accordance with our study. We found a high percentage of subjects with FEI and coronary dilation, but the dimensions of their coronary arteries was smaller than those of subjects diagnosed with KD.

It is known that coronary abnormalities are present in 20% of the cases diagnosed with KD.^16^ In our study, the percentage of coronary dilation among subjects with non-Kawasaki FEI was 26% using the Z score. This implies that coronary changes are more common in FEIs than in KD. It also suggests that it is likely that many of the cases diagnosed as atypical or incomplete KD (based on the presence of coronary alteration) could be, in fact, another FEI.

Pathogenesis of the dilation of coronary arteries in FEIs is not clear. However, it could be related with a greater myocardial demand for oxygen caused by fever and tachycardia. The consequent increase of coronary blood flow is produced through the compensatory dilation of the coronary arteries. Another potential dilation mechanism would involve pathogenic proteins that bind to the endothelial cells, activating immune response pathways that produce cytokines and promote additional cellular damage.

These findings make it clear that the etiology of coronary changes is not unique. There is a common pathophysiological mechanism that can cause temporary or permanent damage. Therefore, coronary changes should be carefully considered for the diagnosis of KD. An echocardiography should be performed in children diagnosed with FEI, and a timely prophylactic treatment should be considered.

Moreover, these findings have implications that should be defined and discussed. Even though the number of subjects is low, the results are important and give rise to some questions:

1. Should an echocardiography be performed to all patients diagnosed with FEIs?
2. If coronary changes are detected, should gamma globulin be administered?
3. Do coronary changes in non-KD FEIs persist or are they reversible?

Any affirmative answer would have an impact on public health and health economics. Maybe, many of the FEI cases primarily considered incompatible with KD should be reconsidered, and the number of the cases of atypical or incomplete KD would increase merely by the fact that the presence of coronary changes is determinant for the diagnosis of non-KD FEI. There is already a similar example in literature: patients without FEI criteria that were diagnosed with KD because of the coronary changes.^17,18^

### Limitations

The main limitation of the study is its small sample size, but despite that, important differences were found. Moreover, it is noteworthy that in our country we do not have nomograms of the coronary arteries of Mexican children, which would allow a direct comparison and avoid the bias inherent to the use of nomograms from other regions. This study can encourage future studies to develop these nomograms with Mexican population. Also, the next step would be to perform a longitudinal study with follow-up of these subjects and assessment of the course of the diseases.

## Conclusions

In this study, we found an important percentage of patients diagnosed with FEI with an alteration in the dimension of the coronary arteries. This makes us conclude that coronary changes acquired in childhood are not exclusive to KD and should be carefully considered when establishing a diagnosis. Although the pathophysiological mechanisms underlying coronary changes in FEIs are not clear, it has been observed that they can cause temporary or permanent coronary damage.
